# Supplementary material for: Whole-Genome Sequence Analysis of Antibiotic Resistance, Virulence, and Plasmid Dynamics in Multidrug-Resistant E. coli Isolates from Imported Shrimp
Source: Foods. 2024 Jun 5;13(11):1766. doi: 10.3390/foods13111766 (PMC11171581; doi:10.3390/foods13111766)
Supplement: Supplementary file 1 [file foods-13-01766-s001.zip › supplementary/foods-3002867-supplementary.pptx]

## Slide 1
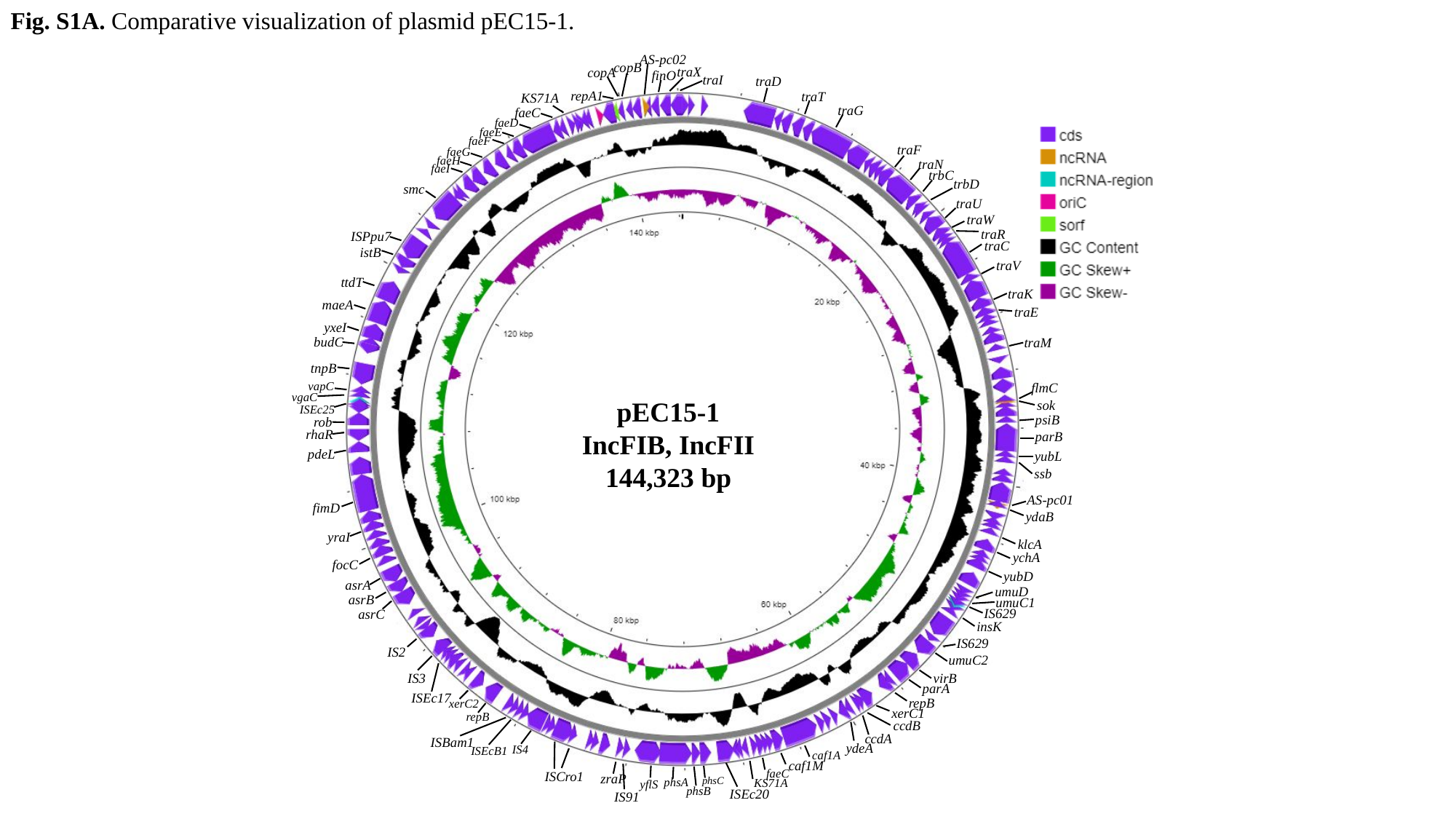

Fig. S1A. Comparative visualization of plasmid pEC15-1.
AS-pc02
copB
traX
copA
finO
traI
traD
repA1
traT
KS71A
traG
faeC
faeD
faeE
faeF
traF
faeG
faeH
traN
faeI
trbC
trbD
smc
traU
traW
traR
ISPpu7
traC
istB
traV
ttdT
traK
maeA
traE
yxeI
budC
traM
tnpB
vapC
flmC
vgaC
pEC15-1
IncFIB, IncFII
144,323 bp
sok
ISEc25
psiB
rob
rhaR
parB
pdeL
yubL
ssb
AS-pc01
fimD
ydaB
yraI
klcA
ychA
focC
yubD
asrA
umuD
asrB
umuC1
IS629
asrC
insK
IS629
IS2
umuC2
IS3
virB
parA
ISEc17
repB
xerC2
xerC1
repB
ccdB
ccdA
ISBam1
ydeA
IS4
ISEcB1
caf1A
caf1M
faeC
ISCro1
zraP
phsC
phsA
KS71A
yflS
phsB
ISEc20
IS91

## Slide 2
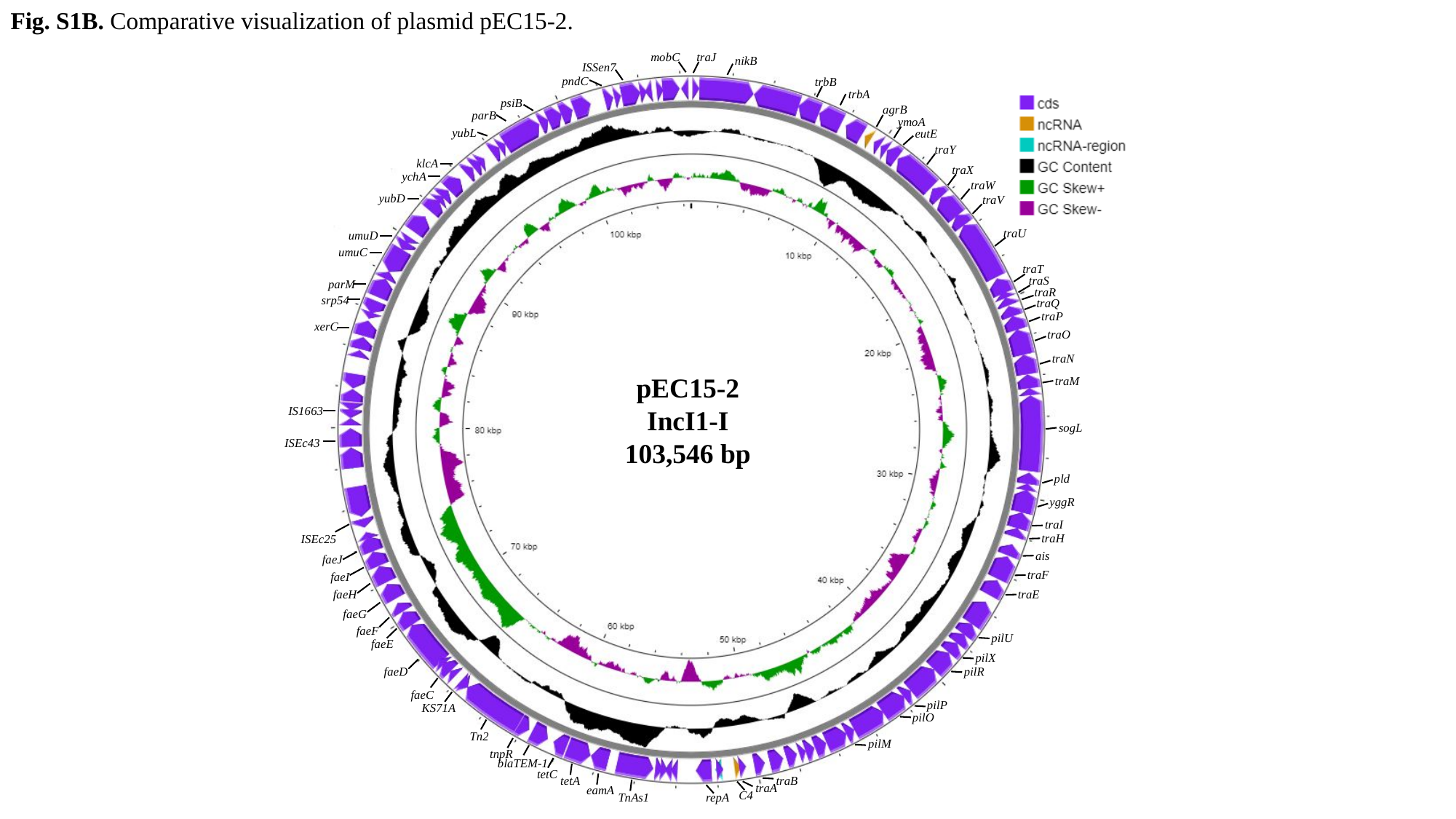

Fig. S1B. Comparative visualization of plasmid pEC15-2.
mobC
traJ
nikB
ISSen7
pndC
trbB
trbA
psiB
agrB
parB
ymoA
yubL
eutE
traY
klcA
traX
ychA
traW
yubD
traV
traU
umuD
umuC
traT
traS
parM
traR
srp54
traQ
traP
xerC
traO
traN
pEC15-2
IncI1-I
103,546 bp
traM
IS1663
sogL
ISEc43
pld
yggR
traI
traH
ISEc25
ais
faeJ
traF
faeI
traE
faeH
faeG
faeF
pilU
faeE
pilX
pilR
faeD
faeC
pilP
KS71A
pilO
Tn2
pilM
tnpR
blaTEM-1
tetC
traB
tetA
traA
eamA
C4
TnAs1
repA

## Slide 3
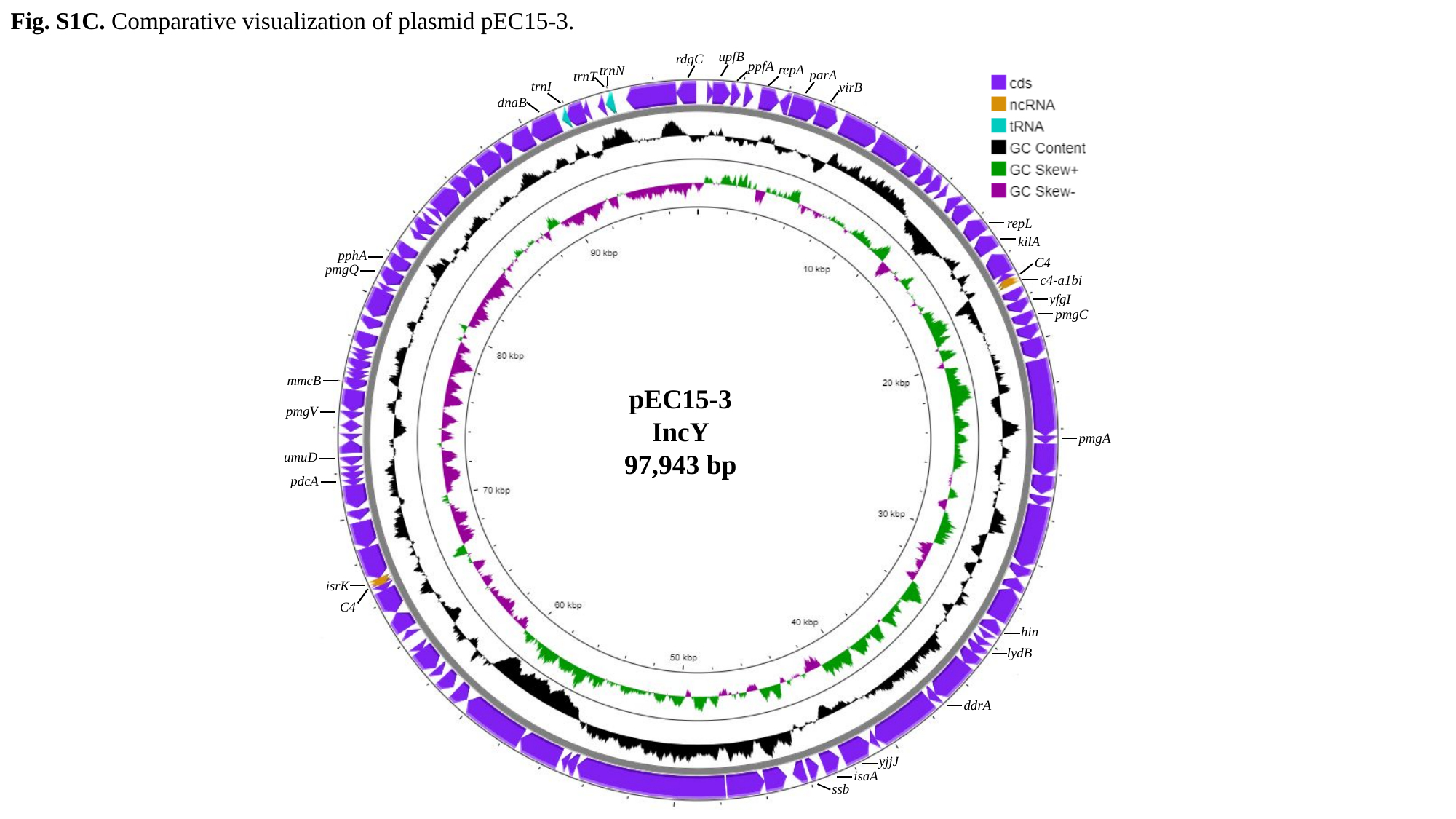

Fig. S1C. Comparative visualization of plasmid pEC15-3.
upfB
rdgC
ppfA
repA
trnN
parA
trnT
trnI
virB
dnaB
repL
kilA
pphA
C4
pmgQ
c4-a1bi
yfgI
pmgC
mmcB
pEC15-3
IncY
97,943 bp
pmgV
pmgA
umuD
pdcA
isrK
C4
hin
lydB
ddrA
yjjJ
isaA
ssb

## Slide 4
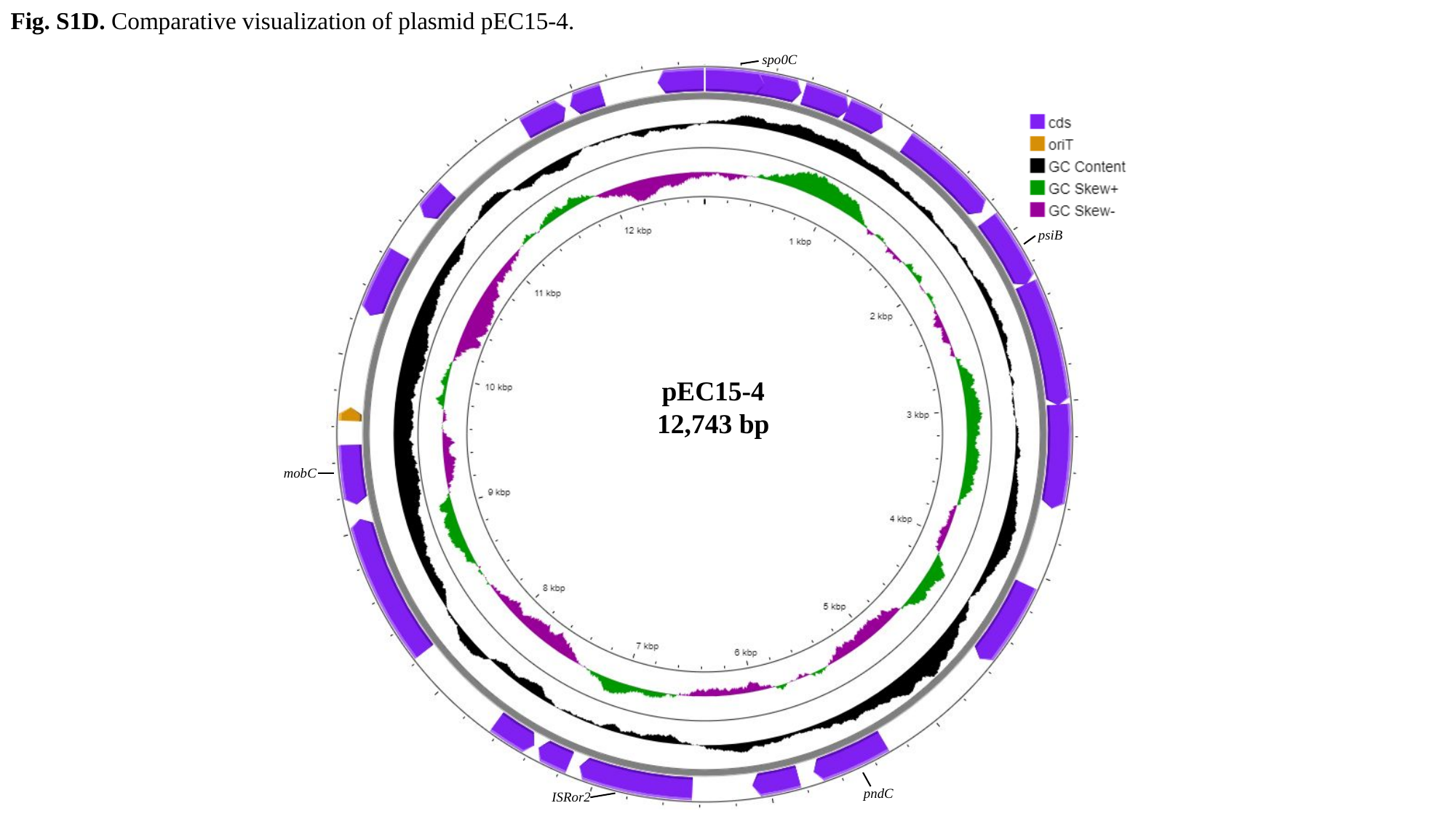

Fig. S1D. Comparative visualization of plasmid pEC15-4.
spo0C
psiB
pEC15-4
12,743 bp
mobC
pndC
ISRor2

## Slide 5
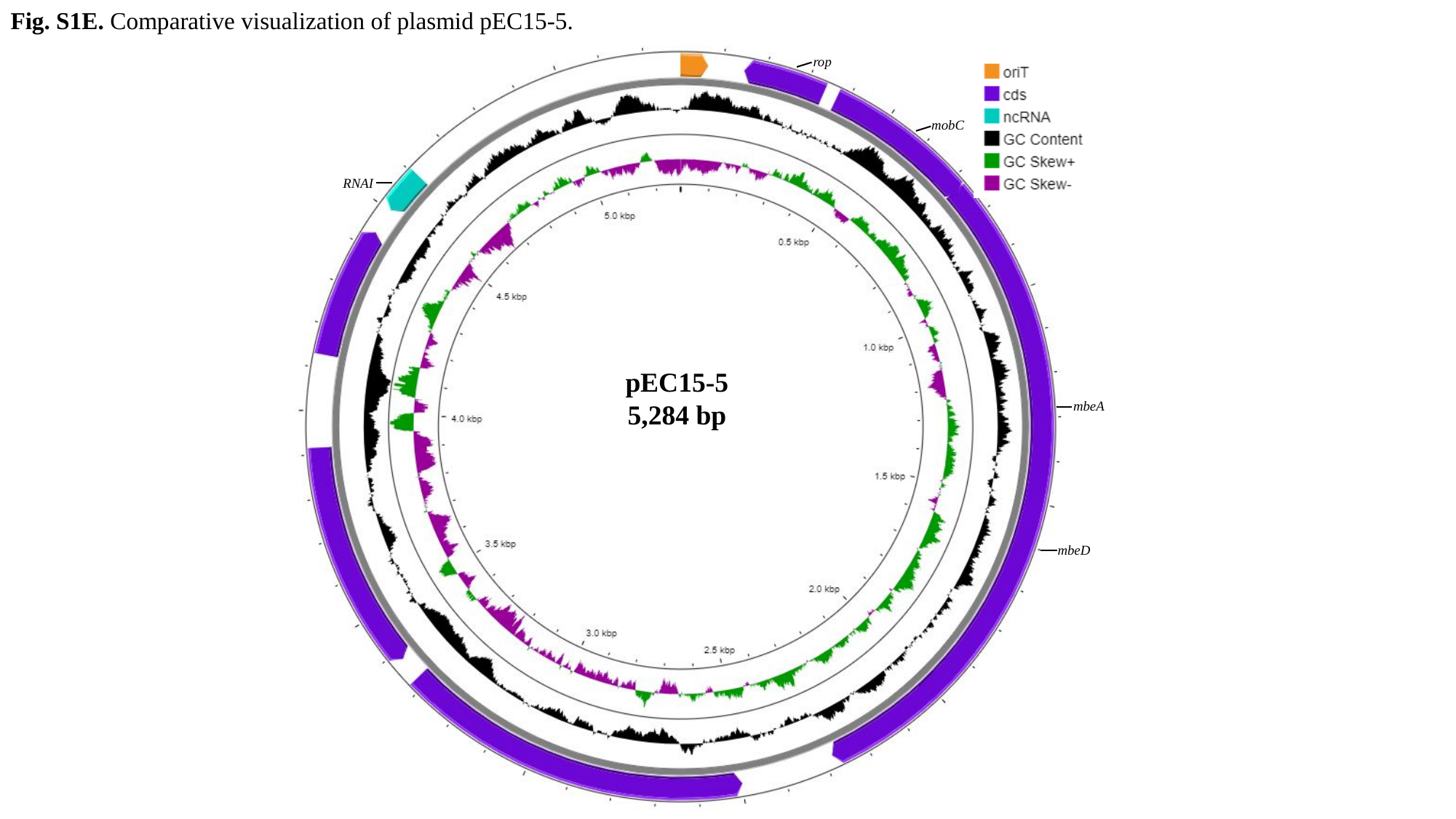

Fig. S1E. Comparative visualization of plasmid pEC15-5.
rop
mobC
RNAI
pEC15-5
5,284 bp
mbeA
mbeD

## Slide 6
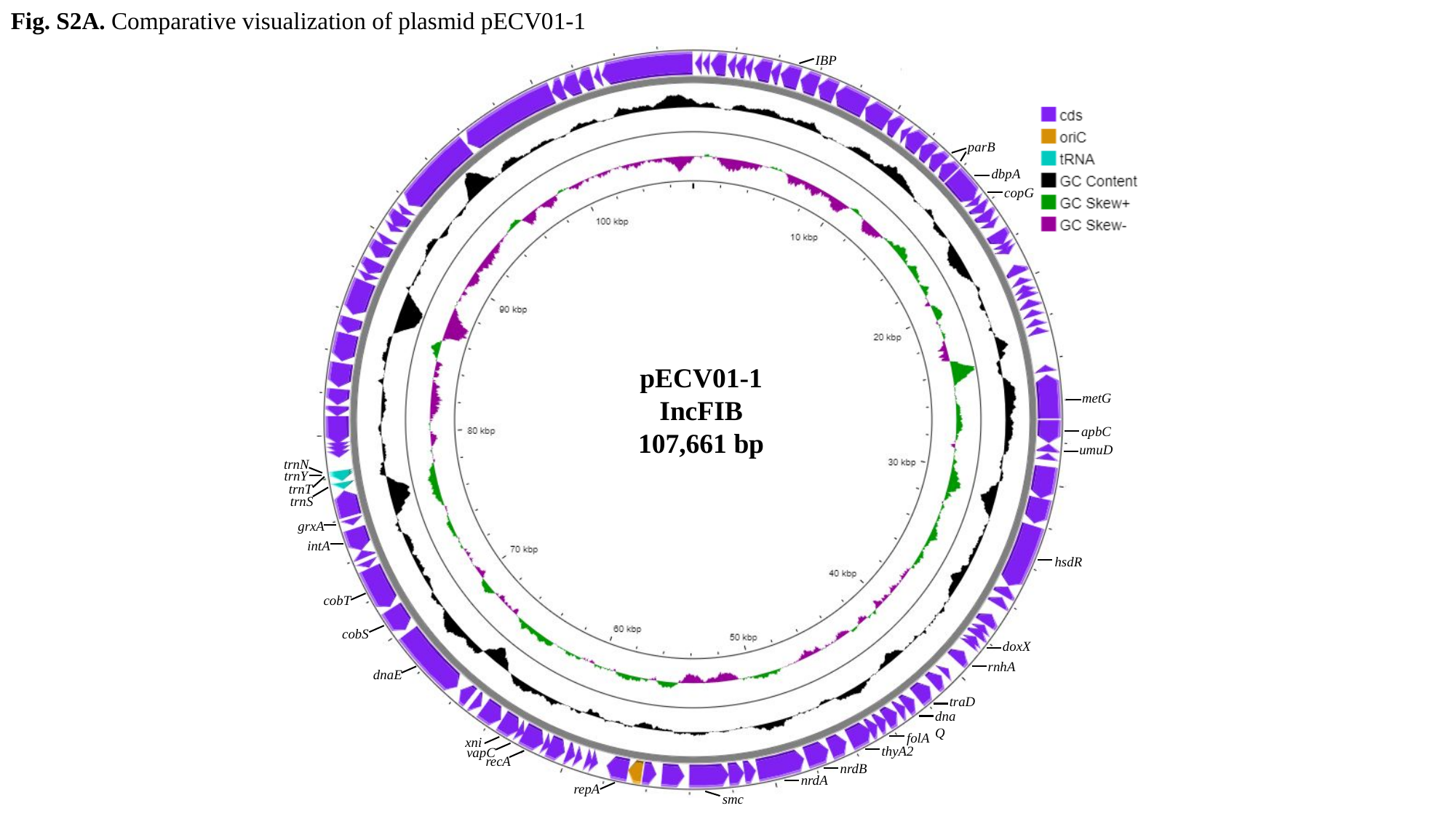

Fig. S2A. Comparative visualization of plasmid pECV01-1
IBP
parB
dbpA
copG
pECV01-1
IncFIB
107,661 bp
metG
apbC
umuD
trnN
trnY
trnT
trnS
grxA
intA
hsdR
cobT
cobS
doxX
rnhA
dnaE
traD
dnaQ
folA
xni
thyA2
vapC
recA
nrdB
nrdA
repA
smc

## Slide 7
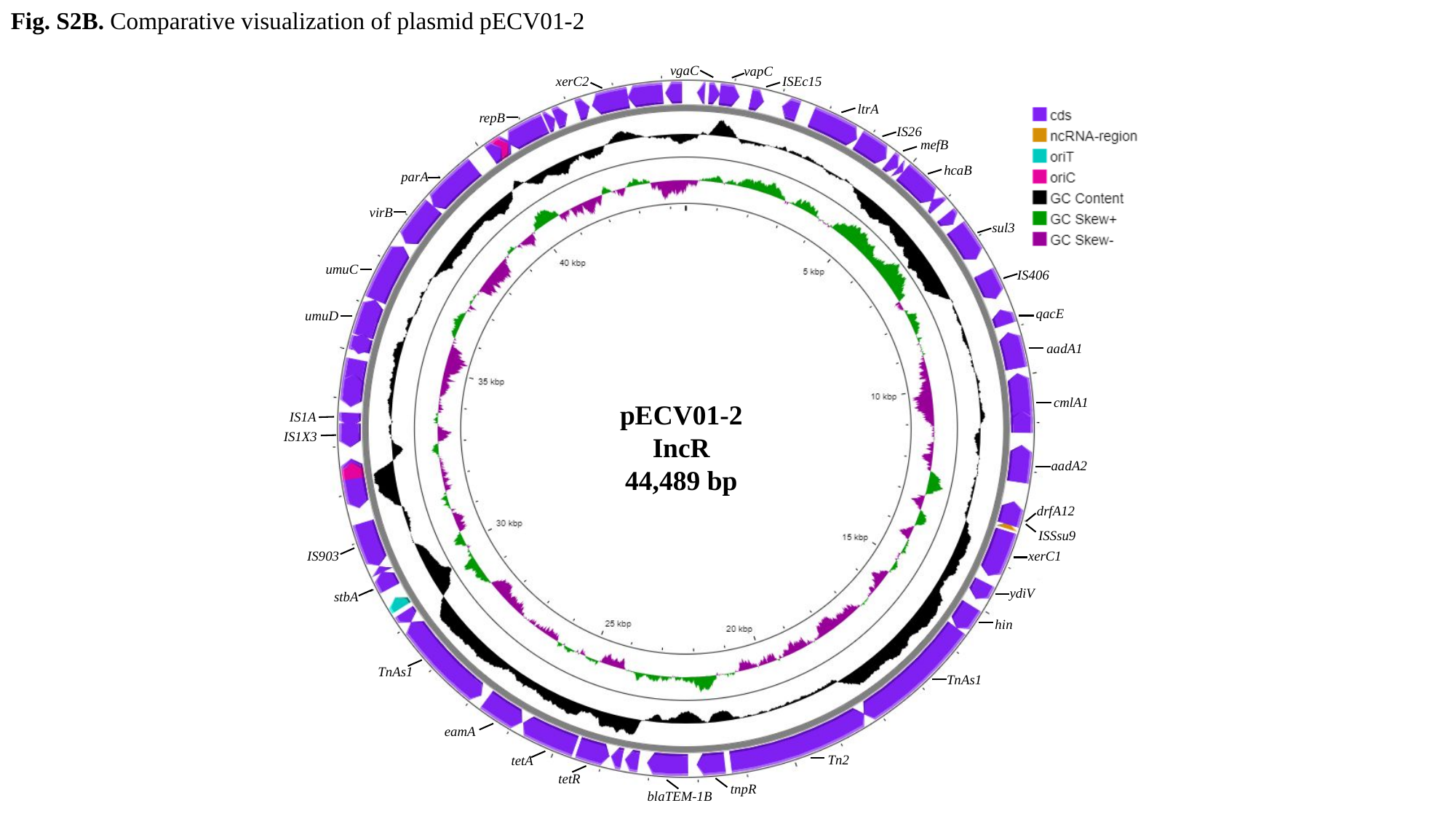

Fig. S2B. Comparative visualization of plasmid pECV01-2
vgaC
vapC
ISEc15
xerC2
ltrA
repB
IS26
mefB
hcaB
parA
virB
sul3
umuC
IS406
qacE
umuD
aadA1
cmlA1
pECV01-2
IncR
44,489 bp
IS1A
IS1X3
aadA2
drfA12
ISSsu9
xerC1
IS903
ydiV
stbA
hin
TnAs1
TnAs1
eamA
Tn2
tetA
tetR
tnpR
blaTEM-1B
